# Neurobehavioral effects of acute low-dose whole-body irradiation

**DOI:** 10.1093/jrr/rrab026

**Published:** 2021-05-13

**Authors:** Mahesh Bekal, Lue Sun, Susumu Ueno, Takashi Moritake

**Affiliations:** Department of Radiobiology and Hygiene Management, Institute of Industrial Ecological Sciences, University of Occupational and Environmental Health, Iseigaoka Yahatanishi-ku, Kitakyushu, Fukuoka 807-8555, Japan; Health and Medical Research Institute, Department of Life Science and Biotechnology, National Institute of Advanced Industrial Science and Technology (AIST), Central 6, 1-1-1 Higashi, Tsukuba, Ibaraki 305-8566, Japan; Department of Occupational Toxicology, Institute of Industrial Ecological Sciences, University of Occupational and Environmental Health, Iseigaoka Yahatanishi-ku, Kitakyushu, Fukuoka 807-8555, Japan; Department of Radiobiology and Hygiene Management, Institute of Industrial Ecological Sciences, University of Occupational and Environmental Health, Iseigaoka Yahatanishi-ku, Kitakyushu, Fukuoka 807-8555, Japan

**Keywords:** total body irradiation, cognitive impairment, anxiety, memory loss, neurotransmitters, low-dose irradiation, acute effect

## Abstract

Radiation exposure has multiple effects on the brain, behavior and cognitive functions. It has been reported that high-dose (>20 Gy) radiation-induced behavior and cognitive aberration partly associated with severe tissue destruction. Low-dose (<3 Gy) exposure can occur in radiological disasters and cerebral endovascular treatment. However, only a few reports analyzed behavior and cognitive functions after low-dose irradiation. This study was undertaken to assess the relationship between brain neurochemistry and behavioral disruption in irradiated mice. The irradiated mice (0.5 Gy, 1 Gy and 3 Gy) were tested for alteration in their normal behavior over 10 days. A serotonin (5-HT), Dopamine, gamma-Aminobutyric acid (GABA) and cortisol analysis was carried out in blood, hippocampus, amygdala and whole brain tissue. There was a significant decline in the exploratory activity of mice exposed to 3 Gy and 1 Gy radiation in an open field test. We observed a significant short-term memory loss in 3 Gy and 1 Gy irradiated mice in Y-Maze. Mice exposed to 1 Gy and 3 Gy radiation exhibited increased anxiety in an elevated plus maze (EPM). The increased anxiety and memory loss patterns were also seen in 0.5 Gy irradiated mice, but the results were not statistically significant. In this study we observed that neurotransmitters are significantly altered after irradiation, but the neuronal cells in the hippocampus were not significantly affected. This study suggests that the low-dose radiation-induced cognitive impairment may be associated with the neurochemical in low-dose irradiation and unlike the high-dose scenario might not be directly related to the morphological changes in the brain.

## INTRODUCTION

Currently radiotherapy and radiosurgery are considered to be the major treatment modalities for various brain disorders such as metastatic neoplasms and arteriovenous malformation, but their benefits are often limited by adverse effects in normal brain tissue. Radiation exposure has multiple effects on the brain, behavior and cognitive functions. These changes depend largely on the dose received and the length of time. Classically, radiation-induced brain injury was attributed to a reduction in the proliferative capacity of glial or vascular endothelial cells [1]. In recent years, it has been observed that patients receiving fractionated partial or whole-brain irradiation (fWBI) can develop significant cognitive impairment, even in the absence of detectable anatomic abnormalities.

The effects of radiation also depend on the dose received. Depending on the animal (rodents) model, a single dose of 10 Gy to the brain can be considered to be under the threshold for vascular changes, demyelination or necrosis, but this dose may be sufficient to inhibit the neural precursor cell proliferation [2]. It has been proposed that changes in neurogenesis play a critical role in cognitive deficit induced by radiation [3]. Populations of hippocampal precursor cell are considerably damaged in a dose dependent manner after whole brain irradiation, even after doses as small as 2 Gy [4,5]. This is most likely because irradiation inhibits neural precursor cell proliferation by disrupting the microenvironment necessary to support neurogenesis [5]. Isono *et al.* showed that low doses of ionizing radiation temporarily arrested the proliferation of mouse neural precursors derived from embryonic stem cells, while cells irradiated at high doses permanently lost their proliferation capability [6]. These differential effects at low- and high-dose radiation exposures are relevant to radiation protection issues as a greater number of individuals are being exposed to low-dose ionizing radiation during diagnostic radiography or occupational activities [7,8].

Before 1970, the human brain was thought to be radioresistant; the acute central nervous system (CNS) syndrome occurs after single doses of ≥30 Gy, and white matter necrosis can occur at fractionated doses of ≥60 Gy. However, it has been established that the CNS is a radiosensitive organ whose degree of dysfunction can be quantified by electrophysiological, biochemical and/or behavioral parameters before the morphological changes appear. Abnormalities in CNS function defined by these parameters may occur at a low dose of whole-body radiation. Therefore, it is important to understand the effects of radiation at low doses.

**Fig. 1.**
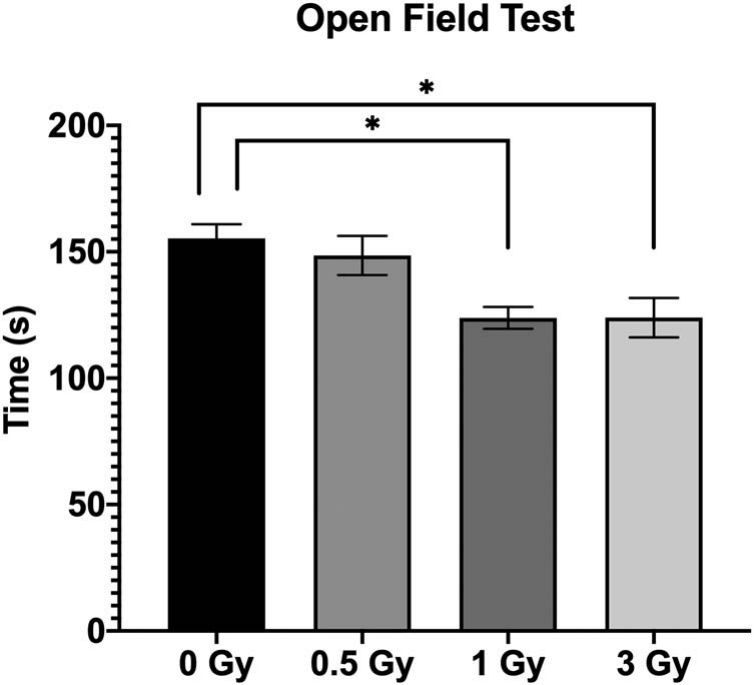
Results of Open Field Test, showing time spent in center of the arena (Mean ± SE, n=6) (^*^P < 0.05; Tukey’s Test).

**Fig. 2.**
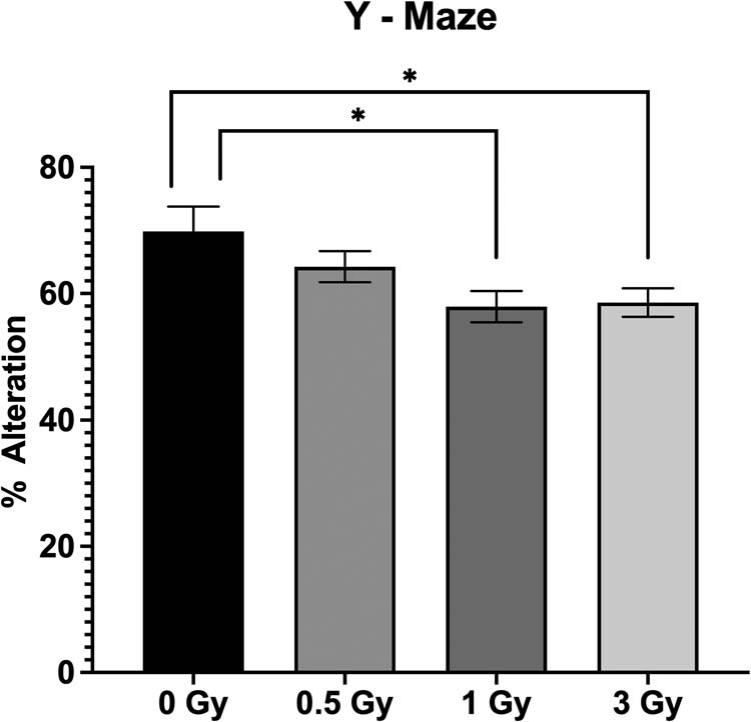
Results of Y-Maze Test, showing the percent alteration (Mean ± SE, n=6) Lower percent alteration indicates the short-term memory loss (^*^P < 0.05; Tukey’s Test).

**Fig. 3.**
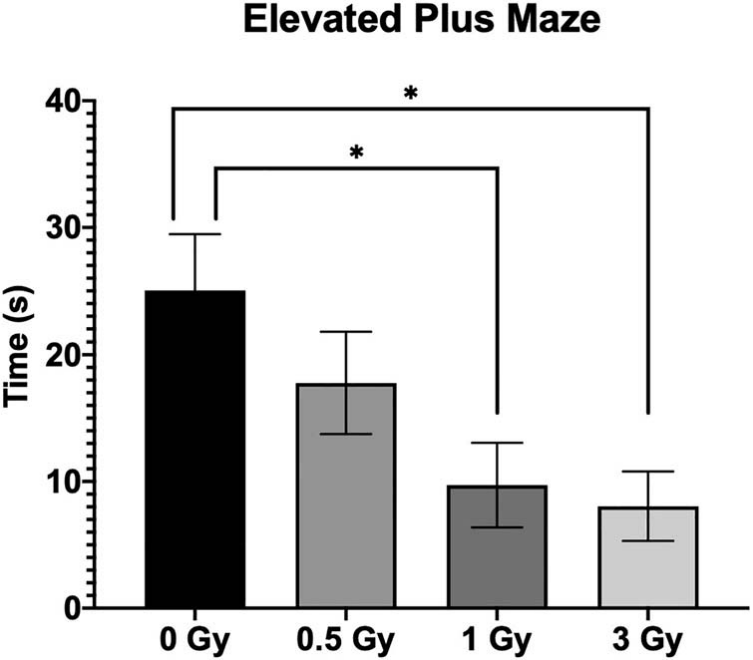
Results of Elevated Plus Maze Test, showing time spent in open arm of the maze(Mean ± SE, n=6). The less time spent in the open arms is indictive of increased anxiety level (^*^P < 0.05; Tukey’s Test).

Although the exact pathogenic mechanisms of low radiation-induced brain injury are not known, a growing body of data suggests that oxidative stress/proinflammatory responses might play a role [9]. Radiation-induced learning and memory deficits in animal models at higher doses are accompanied by a decrease in hippocampal proliferation and a decrease in adult neurogenesis [10,11,12,13]. It is likely that radiation-induced alterations in neurogenesis contribute to cognitive deficits. But effect of low-dose radiation on neuro-behavioral changes is yet to be properly established. This work explores the neurobehavioral changes that occur after the low-dose irradiation and attempts to elucidate the role of neurotransmitters in radiation-induced brain injury.

**Fig. 4.**
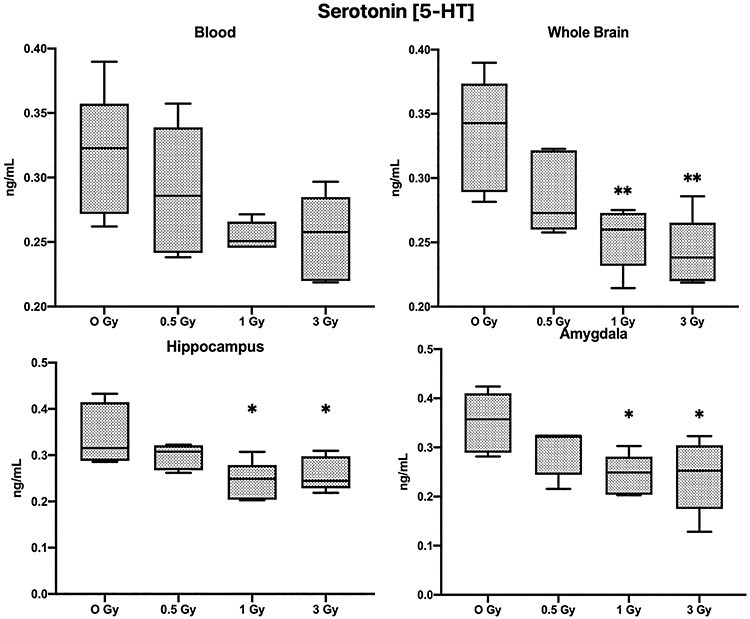
Results of Serotonin (5-HT) analysis (Mean ± SE, n=6), in blood, whole brain tissue, Hippocampus and Amygdala (^*^P < 0.05, ^**^P < 0.01; Tukey’s Test).

## STUDY DESIGN AND METHODS

### Animal and radiation exposure

All animal experiments were performed in accordance with the Animal Care Guidelines of our university and all animal husbandry procedures and experiments were approved by the Institutional Animal Experiment Committee. Animals were divided into four groups, each group contained six- to eight-week-old male C57BL/6J mice (Japan SLC, Shizuoka, Japan). Each group was randomly selected for irradiation. Irradiation was done using the instrument; MBR-1520R-3, Hitachi Power Solutions, Ibaraki, Japan. A dose rate of 0.69 Gy/min was used, a filter of 0.2mm Cu and 0.5mm Al was used to control the precise dose which is consistent with our previous study [14]; Group 1 acted as a sham control (0 Gy), Group 2 received 0.5 Gy, Group 3 received 1 Gy radiation and Group 4 received 3 Gy irradiation. Seven days after irradiation treatment the animals were observed for neurobehavioral alterations using different behavioral paradigms, such as the Open field test, Y-maze and Elevated Plus Maze (EPM). After completion of the behavioral experiments, the animals were sacrificed and the brain was quickly removed and rinsed in in 0.1 M sodium phosphate buffer (pH 7.3) at 4°C and the hippocampus and amygdala region where dissected for the neurotransmitter analysis and randomly selected whole brain was stored in formalin for histopathological analysis. The body weight of the mouse was measured before and after the irradiation and during behavioral studies.

## BEHAVIORAL ANALYSIS

### Circular open field test

The circular open field test (OFT) is a circular enclosure 60 cm in diameter and 50 cm high and the floor is divided into 16 equal grids. The four central grids were considered to be the center, and the rest were assigned as the periphery. The test was performed as previously described [15] and the pertinent behavioral traits were recorded during the time in the arena where the mice were allowed to freely explore for 10 mins, their movements were monitored and recorded using a video camera (Ci-20R, Canon Incorporated, Tokyo). Behavior was analyzed using a behavioral tracing analyzer (BTA-2; Muromachi Instruments Co., Tokyo). Which provides computer-processed data regarding the various activities like time spent in the inner and outer arenas, the latency of moving outside the center circle, the number of lines crossed, total duration of locomotor activity in seconds, total distance moved in centimeters, etc. [15]. This paradigm gives an overview of the affective behavioral functions, such as the exploratory behavior in a novel environment as well as stress and anxiety levels.

### Y-maze

Testing occurred in a Y-shaped maze with three plastic arms at a 120° angle from each other. After introduction to the one arm of the maze, the animal was allowed to freely explore the three arms for 5 mins. Over the course of multiple arm entries, the subject was expected to show a tendency to enter a less recently visited arm. The number of arm entries and the number of triads were recorded in order to calculate the percentage of alternation. This test is used to quantify cognitive deficits (short-term memory) in mice [16].

**Fig. 5.**
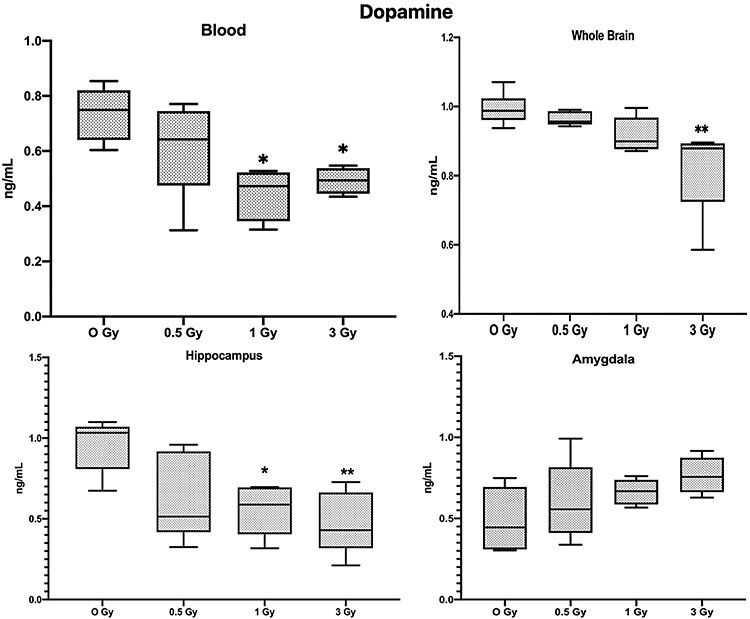
Results of Dopamine analysis (Mean ± SE, n=6), in blood, whole brain tissue, Hippocampus and Amygdala (^*^P < 0.05, ^**^P < 0.01; Tukey’s Test).

### Elevated plus maze

The EPM paradigm assesses measures of anxiety. An EPM consists of two open arms (25 × 5cm) and two (25 × 5cm) closed arms, attached at right angles to a central platform. The apparatus is 40 cm above the floor. The mouse was placed on the central platform with its head towards an open arm. The frequency of entries into the open and closed arm was recorded over 5 mins. In this test, entry into either arm was counted when the mouse had its body and four paws on the arm [17].

### Neurotransmitter analysis

Neurotransmitters in blood and brain tissue were analyzed using commercially available ELISA kits. Serotonin(5-HT), Dopamine was analyzed using kits from ImmuSmol [BA E-5900, BA E-5300], GABA was analyzed using AVIVA Systems Biology Kit [OKEH02564] and Enzo Cortisol ELISA kit [ADI-900-071] was used for the cortisol assay. The assay was conducted according to the method described by the kit manufacturers.

### Histopathological studies

Slices of brain were fixed in 5% formaldehyde, embedded in paraffin wax, sectioned at 5μm thickness and stained with hematoxylin and eosin stain. To estimate radiation-induced brain tissue damage, we measured pyramidal cell density in the dentate gyrus (DG), CA3 and CA1 regions of the hippocampus [18].

### Statistical analysis

Experimental data from all the experiments are expressed as the Mean ± SE. We used one-way analysis of variance (ANOVA) and Tukey’s multiple comparative test to evaluate the statistical difference between the sham and irradiated groups. All the statistical analyses were performed using GraphPad Prism software with a *P* value of <0.05 as significant.

## RESULTS

### Behavioral analysis

In the open field test, there was a significant difference between 0 Gy vs 1 Gy and 3 Gy irradiated mice in the total time spent in the central arena (Fig**.** 1; *P* < 0.05). However, 0.5 Gy did not show any significant changes in the OFT.

Short-term spatial working memory was examined in the Y-maze, the spontaneous alternation was not significant between 0 Gy and 0.5 Gy, But in 0 Gy vs 1 Gy and 3 Gy there was a significant difference in alternation percentage (Fig**.** 2; *P* < 0.05), indicating that 1 Gy and 3 Gy irradiation significantly affect short-term memory.

In the EPM test, 1 Gy and 3 Gy irradiated mice exhibited increased anxiety-like behavior (Fig**.** 3; *P* < 0.05), but in 0.5 Gy irradiated mice the difference was not statistically significant. In all the behavioral tests conducted, although the activity of 0.5 Gy irradiated mice was slightly altered compared with control group, no statistical significance was observed. None of the animals in the study group showed any significant changes in body weight throughout the duration of experiment.

### Biochemical analysis

In irradiated mice, 5-HT levels were significantly decreased in whole brain tissue and also specifically in the hippocampus and amygdala regions, but blood 5-HT levels were not significantly altered (Fig**.** 4; *P* < 0.05).

Dopamine levels were significantly decreased in the blood of 3 Gy and 1 Gy irradiated mice (Fig**.** 5; *P* < 0.05), and 3 Gy irradiated mice showed a significant decrease in whole brain tissue and hippocampus, whereas 1 Gy mice showed a significant decrease in the hippocampus only.

**Fig. 6.**
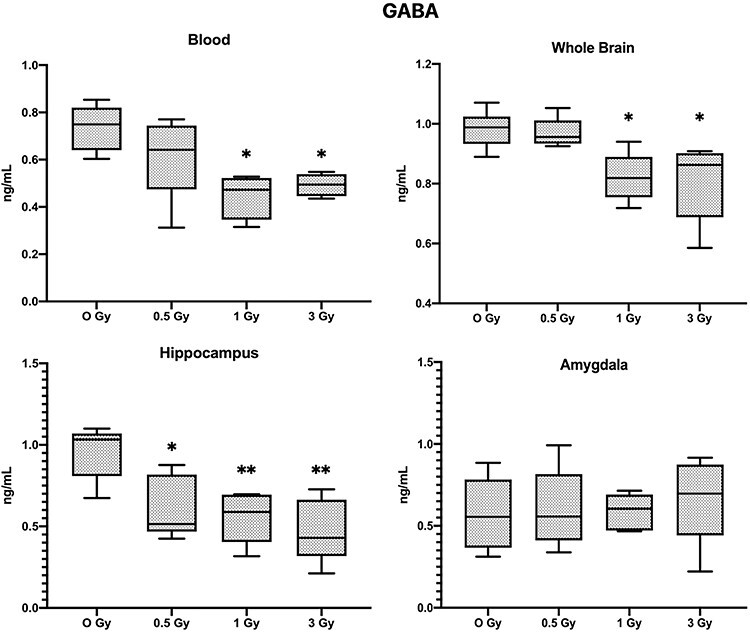
Results of GABA analysis (Mean ± SE, n=6), in blood, whole brain tissue, Hippocampus and Amygdala (^*^P < 0.05, ^**^P < 0.01; Tukey’s Test).

GABA levels were significantly decreased in whole brain tissue and also specifically in the hippocampus and but amygdala regions did not show any statically significant changes, however, blood GABA levels were significantly altered in the 1 Gy and 3 Gy irradiated groups (Fig**.** 6, *P* < 0.05). whereas 0.5 Gy irradiated mice showed a significant decrease of GABA levels in the hippocampus region only.

**Fig. 7.**
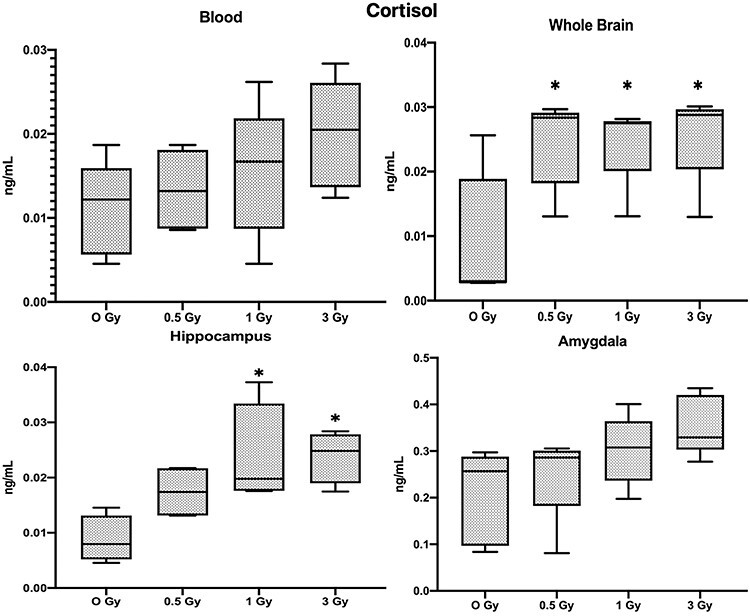
Results of Cortisol analysis (Mean ± SE, n=6), in blood, whole brain tissue, Hippocampus and Amygdala (^*^P < 0.05, ^**^P < 0.01; Tukey’s Test).

No significant changes in blood cortisol level were observed, but all the irradiated groups including 0.5 Gy showed significant increases of cortisol in whole brain tissue (Fig**. 7**; *P* < 0.05). But in the hippocampus only 1 Gy and 3 Gy showed a significant increase and in the amygdala there was no significant change to the cortisol levels in any of the study groups. (Detailed statistical analysis of neurotransmitters is given in Supplementary Table S1)

### Histopathological analysis

**Fig. 8.**
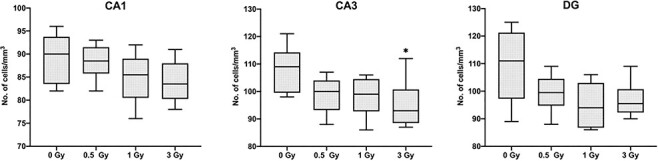
Results of Histopathological analysis. Number of pyramidal cells (Mean ± SE, n=6) in CA1, CA3 and DG regions of Hippocampus, (^*^P < 0.05, ^**^P < 0.01; Tukey’s Test).

Only the group irradiated at 3 Gy showed a significant decrease in cells in the CA3 region of the hippocampus, and in CA1 and DG 3 Gy irradiated mice showed no changes compared to the control group. There were no significant changes in the DG, CA3 and CA1 regions of the 1 Gy and 0.5 Gy irradiated mice. (Fig**.** 8; *P* < 0.05).

## DISCUSSION

We have conducted many trials of behavioral studies at one, three, seven, 10 and 30 days after irradiation (data not shown), and observed that results were highly consistent and reproducible at seven to 10 days after irradiation, so we chose seven to 10 days as the optimal time point to conduct the experiments. The current results indicated that acute (seven to 10 days) whole-body X-ray irradiation of 1 Gy and 3 Gy in mice can lead to cognitive deterioration. In addition, irradiation in mice showed a number of alterations to the neurotransmitter levels, but the hippocampus cell density was not significantly altered. There are number of studies that suggest that the cognitive deterioration caused by the radiation is due to the disruption of hippocampal neurogenesis and neural precursor cells of the DG are very sensitive to irradiation and are not capable of repopulating the sub granular zone (SGZ) at least up to 120 days after irradiation at doses of 5 Gy or higher [10,19]. It is likely that radiation-induced alterations in neurogenesis contribute to cognitive deficits, but our study showed that the cognitive dysfunction occurs at lower doses like 3 Gy and 1 Gy and in this study we did not observe the depletion of neuronal cells in CA1, CA3 and DG region of the 1 Gy irradiated mice, this suggests that at lower doses behavioral alterations may be mediated by some other mechanisms. To identify the mechanism of behavioral disruptions in irradiated mice, we analyzed the neurotransmitters in these mice, we quantified levels of neurochemicals like Dopamine, GABA, 5-HT and stress markers such as cortisol in the blood and several brain regions. We specifically focused on the hippocampus and amygdala because the hippocampus is important for memory consolidation and learning behavior [20] and the amygdala is involved primarily in emotional responses like anxiety, stress and fear conditioning [21,22].

One of the most notable molecules for the pathophysiology of depression and anxiety is 5-HT [23]. In irradiated mice, 5-HT levels were significantly decreased in whole brain tissue and also specifically in the hippocampus and amygdala regions, but blood 5-HT levels were not significantly altered. In contrast, Dopamine levels in the blood of the irradiated mice were significantly decreased but there was no change in levels of the amygdala region, but it was significantly changed in the hippocampus. Dopamine is linked to the regulation of motivation, psychomotor speed, concentration and the ability to experience pleasure [24]. The decrease in exploratory activity, anxiety-like behavior and impaired memory of irradiated animals in our study could also be attributed to decreased dopamine levels in the hippocampus, since normal dopamine functioning is to be required for the establishment and maintenance of incentive learning in animals [25]. In our study GABA levels were also decreased in blood, whole brain and the hippocampus of the irradiated mice, Over recent years many preclinical and clinical studies have accumulated evidence suggesting that a GABA deficit may be involved in mood disorders, particularly in depression-like symptoms [26].

Cortisol is a glucocorticoid hormone that is synthesized and secreted by the cortex of adrenal glands. In this study we observed the increased cortisol levels in the whole brain and hippocampus. The relationship between stress, elevated levels of cortisol and hippocampal structure and memory deficits has been a topic of intense discussion; cortisol elevation causes damage to the hippocampus and impairs hippocampus-dependent learning and memory [27]. Chronic high cortisol causes functional atrophy of the hypothalamic-pituitary-adrenal axis. Increased cortisol levels are related to a number of psychosocial factors, like major depression, stress and a variety of cognitive processes [28].

The decreased brain 5-HT and elevated cortisol (glucocorticoid) secretion in major depression syndromes is a well-established fact. More recent experiments have suggested that elevated cortisol levels, due to stress, may lead to lower brain 5-HT function and in turn lead to the manifestation of a depressive state [29]. These findings suggest that the cognitive changes in the brains of irradiated mice might be modulated by the neurochemical differences in the brain.

Radiation-induced cognitive changes, which are believed to be caused by the destruction of neural stem cells in the hippocampus region of the brain, a study by Mizumatsu *et al.* [30] showed that the cells of SGZ of the hippocampus are extremely sensitive to X-rays and hippocampal neurogenesis is altered in a dose dependent manner. Given the role of the hippocampus in specific cognitive functions, their study supported the idea that changes in hippocampal neurogenesis may play an important role in radiation-induced cognitive impairment. Our results observed that at higher doses of 3 Gy the neuronal cell of CA3 regions of the hippocampus are reduced, but at lower doses of 1 Gy hippocampal cells are intact, but cognitive functions were still affected. This finding suggests that low-dose radiation might be affecting the normal neuronal functions like production and regulation of neurotransmitters through some other mechanism and ultimately affecting higher brain function. Hritcu *et al.* [31] showed that the generalized depletion of 5-HT in the brain produces an impairment of short-term memory, our study revealed that the irradiated mice exhibited impaired short-term memory in the Y-maze, and also there was a significant decrease of 5-HT in the hippocampus and amygdala, as well as the whole brain tissue of irradiated mice, this might be an indication of the involvement of the serotogenic system in radiation-induced memory deficit. In this study it is evident that neurotransmitters like 5-HT, dopamine and GABA are significantly altered after irradiation. These results, coupled with an earlier study conducted by our research group in which we discovered that radiation significantly altered the neurotransmitters related metabolites [32], strongly suggest that the neurotransmitters play a significant role in the mechanism of low-dose radiation-induced cognitive deterioration.

Many neurotransmitter levels in the brain showed a similar pattern as the blood. These are interesting phenomena, and we believe that the neurotransmitter levels in the blood have the potential to become the indicator for the prediction of brain neurotransmitter levels, as well as potential biomarker for radiation-induced cognitive changes. However, further study in this area is required to understand how these neurotransmitter levels are altered, and the sensitivity of these potential biomarkers needs to be studied in detail. While the neurotransmitters like 5-HT and GABA follow the trend of dose dependent decrease, we observed that dopamine levels in the amygdala contradict this pattern, i.e. Dopamine levels in the amygdala slightly increase with the dose (results are statistically not significant), but dopamine in blood, hippocampus and whole brain tissue decreased significantly. Some imaging studies have shown that the stress can induce changes to the amygdala but not to the hippocampus [33], and some studies have revealed that the neuronal circuits for reward and fear memory formation and mood regulation involve the amygdala through a dopaminergic connection, and memory formation is modulated by hippocampus through GABAergeic pathway. However, overall mood and depression-like behaviors are regulated by the intervention of several brain regions by monoaminergic (e.g. serotonin and GABA) inputs [34]. We suspect that interaction between the hippocampus and amygdala might have a bigger role in the radiation-induced cognitive changes. Overall it could be said that the brain is a complex organ and the normal functioning of the brain involves various structures like the hippocampus, amygdala and a communication network of various neurotransmitters. Between them, these structures could be a key element to aid understanding of the neurocognitive alterations induced by radiation.

## CONCLUSION

Brain radiation injury is multifactorial and complex and is characterized by a range of molecular/cellular/tissue alterations. A number of investigations about the effects of radiation on the brain have been carried out. However, these have been investigated mostly in high dose/dose rate models, and mainly focus on a neurogenesis, inflammation and oxidative stress model, whereas our study has a unique approach. This study suggests that the low-dose radiation-induced cognitive impairment may be associated with the neurochemical in low-dose irradiation, and unlike the high-dose scenario, might not be directly related to morphological changes in the brain. Further investigation in this direction may help to identify the specific mechanism involved in the radiation-induced neurobehavioral alteration, and ultimately elucidation of the involved cellular and molecular mechanisms will help in the development of new preventive and therapeutic methods, mitigating the adverse effects of brain irradiation.

## Supplementary Material

Supplementary_Table_1_rrab026
