# Supplementary material for: Neurobehavioral effects of acute low-dose whole-body irradiation
Source: J Radiat Res. 2021 May 13;62(5):804–11. doi: 10.1093/jrr/rrab026 (PMC8438260; doi:10.1093/jrr/rrab026)
Supplement: Supplementary_Table_1_rrab026 [file supplementary_table_1_rrab026.docx]

Table 1. Results of Tukey's multiple comparisons test analysis of 5-HT, Dopamine, GABA and Cortisol. Showing the p value between the groups, P value <0.05 is significant.

| **Tukey's multiple**  **comparisons test** | | **O Gy vs. 0.5 Gy** | **O Gy vs. 1 Gy** | **O Gy vs. 3 Gy** |
| --- | --- | --- | --- | --- |
| **Serotonin** | Blood | 0.7084 | 0.1048 | 0.0951 |
|  | Whole Brain | 0.1549 | 0.0070 | 0.0021 |
|  | Hippocampus | 0.3987 | 0.0146 | 0.0452 |
|  | Amygdala | 0.3951 | 0.0429 | 0.0392 |
| **Dopamine** | Blood | 0.4268 | 0.0141 | 0.0410 |
|  | Whole Brain | 0.9184 | 0.3605 | 0.0071 |
|  | Hippocampus | 0.0708 | 0.0191 | 0.0049 |
|  | Amygdala | 0.06929 | 0.3573 | 0.0671 |
| **GABA** | Blood | 0.4286 | 0.0141 | 0.0410 |
|  | Whole Brain | 0.9946 | 0.0306 | 0.0185 |
|  | Hippocampus | 0.0253 | 0.0080 | 0.0017 |
|  | Amygdala | 0.9965 | 0.9997 | 0.9016 |
| **Cortisol** | Blood | 0.9362 | 0.6741 | 0.1525 |
|  | Whole Brain | 0.0301 | 0.0293 | 0.0188 |
|  | Hippocampus | 0.2454 | 0.0231 | 0.0206 |
|  | Amygdala | 0.8851 | 0.3403 | 0.0567 |
